# Adverse arrhythmia-related outcomes after catheter ablation in patients with inflammatory bowel disease: a nationwide propensity score–matched cohort study

**DOI:** 10.1093/ehjopen/oeag168

**Published:** 2026-10-08

**Authors:** Ramin Ebrahimi, Shaojie Chen, Davide Di Vece, Michele Maremmani, Ali Mahdi, Bahira Shahim, Jan Hendrik Niess, Alexander Kutz, Fahim Ebrahimi, Anders Forss

**Affiliations:** Division of Cardiology, Department of Internal Medicine B, University Medicine Greifswald, Fleischmannstrasse 10, 174 75 Greifswald, Germany; Division of Cardiology, Department of Internal Medicine B, University Medicine Greifswald, Fleischmannstrasse 10, 174 75 Greifswald, Germany; Division of Cardiology, Department of Internal Medicine B, University Medicine Greifswald, Fleischmannstrasse 10, 174 75 Greifswald, Germany; First Clinic of Internal Medicine, Department of Internal Medicine, University of Genoa, 6 viale Benedetto XV, 161 32 Genoa, Italy; Department of Cardiology, Pio XI Hospital, ASST Brianza, Via Guiseppe Mazzini 1, 208 32 Desio, Italy; Division of Cardiology, Department of Medicine Solna, Karolinska Institutet, Norrbacka S1:02, Karolinska University Hospital, 171 76 Stockholm, Sweden; Heart and Vascular Theme, Karolinska University Hospital, Eugeniavägen 3, 171 76 Stockholm, Sweden; Division of Cardiology, Department of Medicine Solna, Karolinska Institutet, Norrbacka S1:02, Karolinska University Hospital, 171 76 Stockholm, Sweden; Heart and Vascular Theme, Karolinska University Hospital, Eugeniavägen 3, 171 76 Stockholm, Sweden; Gastroenterology Group, Department of Biomedicine, University of Basel, Petersgraben 4, 4031 Basel, Switzerland; Department of Gastroenterology and Hepatology, University Digestive Health Care Center Basel–Clarunis, Petersgraben 4, 4031 Basel, Switzerland; Division of General Internal Medicine, University Department of Medicine, Kantonsspital Aarau, Tellstrasse 25, 5001 Aarau, Switzerland; Division of Pharmacoepidemiology and Pharmacoeconomics, Department of Medicine, Brigham and Women’s Hospital and Harvard Medical School, 1620 Tremont street, Suite 3030, Boston, MA 02120, USA; Medical Faculty of the University of Basel, Klingelbergstrasse 61, 4056 Basel, Switzerland; Department of Gastroenterology and Hepatology, University Digestive Health Care Center Basel–Clarunis, Petersgraben 4, 4031 Basel, Switzerland; Medical Faculty of the University of Basel, Klingelbergstrasse 61, 4056 Basel, Switzerland; Department of Medical Epidemiology and Biostatistics, Karolinska Institutet, Nobels väg 12 A, 171 65 Stockholm, Sweden; Centre for Digestive Health, Department of Gastroenterology, Dermatovenereology and Rheumatology, Karolinska University Hospital, Eugeniavägen 3, 171 76 Stockholm, Sweden; Department of Molecular Medicine and Surgery, Karolinska institutet, Karolinska University Hospital, Solna (L1:00), 171 76 Stockholm, Sweden

**Keywords:** Ablation, Arrhythmia, Epidemiology, Inflammatory bowel disease

## Abstract

**Aims:**

Inflammatory bowel diseases (IBD) have been associated with an increased long-term risk of arrhythmia. However, clinical outcomes following catheter ablation in patients with IBD are unknown.

**Methods and results:**

In this nationwide Swiss cohort study of adults undergoing catheter ablation for arrhythmia between 2012 and 2022, patients with IBD were propensity score–matched 1:10 to comparators without IBD. The primary endpoint was a composite of the first occurrence of any of the following events: reablation, cardioversion, or hospitalization for arrhythmia. Secondary endpoints were each individual component of the primary endpoint. An additional win ratio-based hierarchical analysis was performed. Hazard ratios (HRs) and incidence rates (IRs) were calculated using Cox proportional hazards modelling. A total of 251 patients with IBD and 2429 matched comparators were included in the study. After a median follow-up time of 2.6 years (IQR 0.8–5.6), the primary endpoint occurred in 81 (32.3%, IR: 100.8/1000 person-years) patients with IBD and in 640 (26.3%, IR: 73.3/1000 person-years) matched comparators, yielding an HR of 1.30 [95% confidence interval (CI) = 1.03–1.64]. Among the individual components of the primary endpoint, hospitalization for arrhythmia was more frequent in patients with IBD (HR 1.29, 95% CI = 1.02–1.64), while the remaining components did not differ between groups. In hierarchical win ratio analysis, patients with IBD had worse composite outcomes than matched comparators (win ratio 0.80, 95% CI = 0.65–0.99, *P* = 0.04).

**Conclusion:**

Patients with IBD undergoing catheter ablation exhibited 30% higher hazards of adverse arrhythmia-related outcomes.

## Graphical Abstract

**Graphical Abstract:**
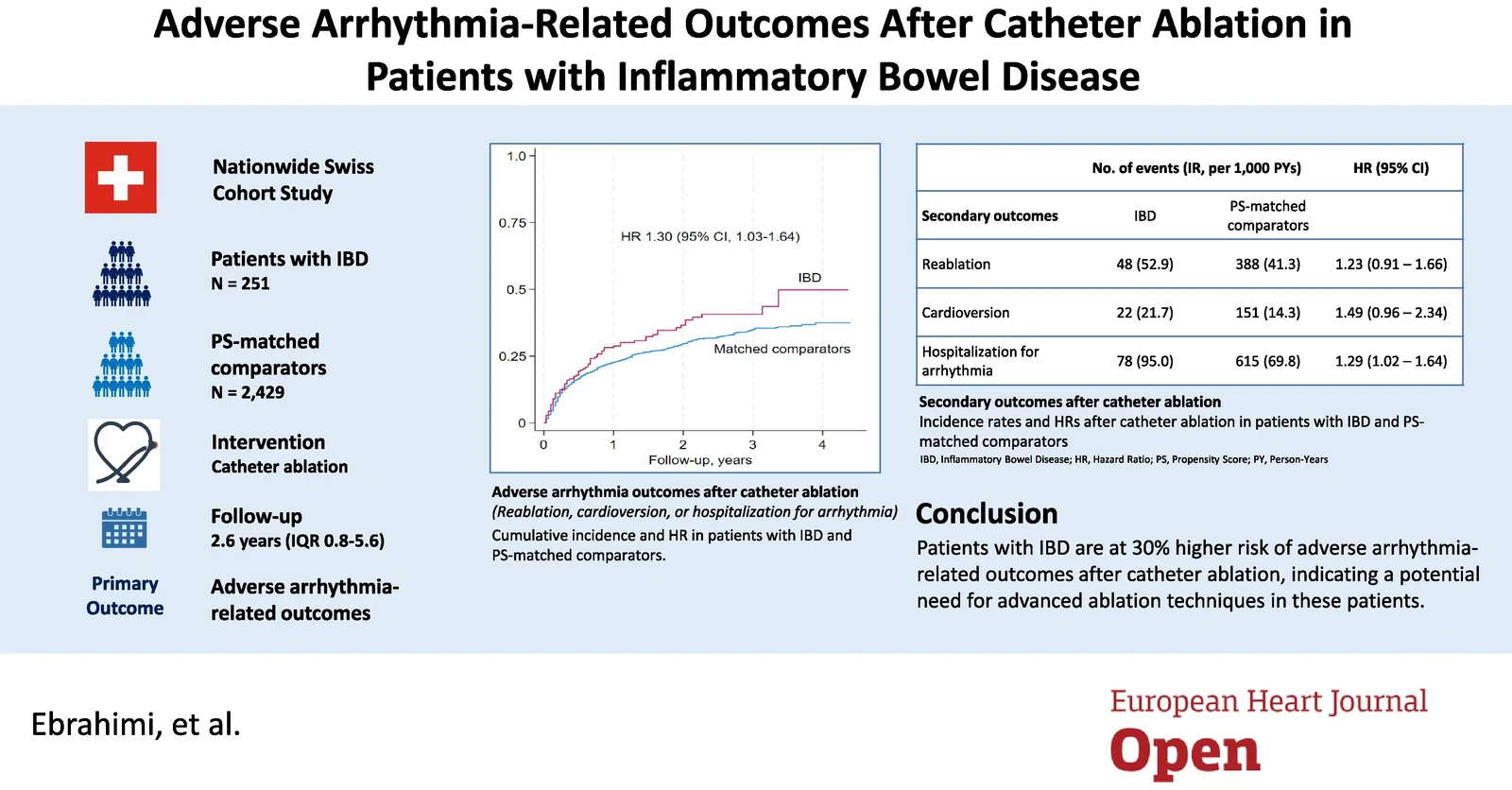

Translational perspective Despite the explorative nature of this study, the findings may suggest that IBD identifies a subgroup of patients undergoing catheter ablation who may potentially be at increased risk of post-procedural recurrence and other arrhythmia-related outcomes. Although causal mechanisms cannot be established in our study, chronic systemic inflammation and disease-related factors may play a contributory role in this association, as has been proposed for other adverse cardiovascular outcomes in IBD. If our findings are confirmed in future studies, enhanced clinical vigilance, careful cardiovascular risk assessment, and close post-ablation follow-up may be warranted in patients with IBD. Indeed, further studies incorporating data on IBD disease activity, treatment exposure, arrhythmia burden, and granular procedural details are needed to clarify the underlying mechanisms and to inform any potential need for optimized management strategies in this population.

## Introduction

Inflammatory bowel diseases (IBD), including Crohn’s disease (CD) and ulcerative colitis (UC), are chronic, immune-mediated inflammatory disorders of the gastrointestinal tract.^1,2^ The global prevalence of IBD has increased substantially, currently affecting ∼0.5–1% of the population in high-income countries.^3^

Chronic systemic inflammation, a hallmark of IBD, plays a key role in the pathogenesis and progression of atherosclerosis.^4^ Accordingly, multiple studies have reported an increased long-term risk of cardiovascular disease (CVD) among patients with IBD.^5–7^ Additionally, recent findings have shown that patients with IBD appear to have a higher risk of recurrent cardiovascular events following coronary interventions compared with the general population.^8^ Systemic inflammation has also been implicated in arrhythmogenicity through mechanisms including oxidative stress, autonomic dysfunction, and electrolyte imbalances.^9–13^ Hence, IBD has been associated with an increased long-term risk of arrhythmia^5–7^; however, it remains unclear whether patients with IBD are also at heightened risk of recurrent adverse arrhythmia-related outcomes following catheter ablation. Given the potential role of chronic systemic inflammation in arrhythmogenesis and post-procedural outcomes, clarifying this association is clinically relevant for optimizing ablation strategies and post-ablation care in this population.

Therefore, in this nationwide matched cohort study of individuals undergoing catheter ablation, we aimed to assess whether IBD is associated with an increased risk of adverse arrhythmia-related outcomes.

## Methods

This nationwide cohort study utilized fully pseudo-anonymized, population-based administrative claims data from the Swiss Federal Statistical Office (Bundesamt für Statistik, Neuchâtel, Switzerland). The database includes all Swiss inpatient discharge records from acute care and specialty hospitals, excluding post-acute care units, regardless of payer, thereby providing a comprehensive population-based sample of inpatient discharges in Switzerland. Patient-level data on demographics, healthcare utilization, hospital type, discharge diagnoses, clinical procedures, and in-hospital outcomes, including length of stay (LOS), intensive care unit (ICU) admission, intubation, and in-hospital mortality, were available for all hospitalized patients. Each individual was uniquely identified using an anonymous identifier, which enabled longitudinal follow-up and the tracking of re-hospitalizations and re-interventions. Medical diagnoses were coded according to the International Classification of Diseases, 10th Revision, German Modification (ICD-10-GM), and procedures were coded using the Swiss Classification of Operations codes.^14^ This study was conducted in accordance with the principles of the Declaration of Helsinki^15^ and adhered to the Strengthening the Reporting of Observational Studies in Epidemiology (STROBE) guidelines.

### Ethics

The institutional review board of Northwestern Switzerland approved this study (Req-2021-01397) and granted a waiver of informed consent due to the use of de-identified data.

### Study population and exposure definition

All adults who underwent cardiac catheter ablation in Switzerland between 1 January 2012 and 31 December 2022 were eligible for inclusion. Catheter ablation for atrial or ventricular arrhythmias was identified using hospital discharge procedure codes 37.34.XX (where X denotes wildcard characters; Supplementary material online, *Table S1*). Individuals with IBD were identified using ICD-10-GM codes K50 and K51 (see Supplementary material online, *Table S2*). Patients younger than 20 years and admissions from non-somatic hospitals were excluded. Patients with IBD were propensity score–matched with individuals from the general population without IBD (see Supplementary material online, *Figure S1*). Follow-up began the day after hospital discharge and continued until the occurrence of any study outcome, death, or the end of the study period on 31 December 2022.

### Study outcomes

The primary composite endpoint was arrhythmia-related adverse outcomes, defined as reablation, cardioversion, or hospitalization for arrhythmia occurring at any time after index catheter ablation (see Supplementary material online, *Table S3*). Secondary endpoints included each individual component of the primary endpoint, 4-point major adverse cardiovascular events (MACE), defined as a composite of acute myocardial infarction, stroke, hospitalization for heart failure, and all-cause death, as well as 30-day all-cause readmission rate, and in-hospital outcomes including ICU admission, length of ICU stay, need for intubation, duration of intubation, and length of LOS (see Supplementary material online, *Table S3*). For patients transferred between acute care hospitals, hospital stays were combined into a single episode of care, with outcomes attributed to the initial hospitalization. Any re-hospitalization within 30 days of discharge was classified as a readmission for the all-cause readmission rate outcome. The analysis of readmissions included both emergency and planned hospital admissions.

### Statistical analysis

Descriptive statistics were used to summarize baseline characteristics. Continuous variables are reported as means and standard deviations (SD) and categorical variables as counts and percentages. Standardized mean differences (SMD) were calculated to assess differences between the two groups; values ≥ 0.10 indicated meaningful imbalance.^16^ No data were missing for patient characteristics or study outcomes.

To account for heterogeneity within the study cohort, we conducted a propensity score–matching using a logistic regression model that included all relevant, documented comorbidities up to and including the index date (defined as the date of index catheter ablation). This approach was used to balance baseline characteristics between patients with IBD and matched comparators from the general population, thereby reducing measurable confounding in this observational analysis. Eligible patients with IBD were matched to comparators in a 1:10 ratio using nearest-neighbour matching with replacement, based on predefined inclusion and exclusion criteria. The distribution of propensity scores before and after matching was assessed graphically (see Supplementary material online, *Figure S2*).

To further explore our findings, we conducted stratified analyses of clinical outcomes after catheter ablation based on the indication for the index procedure [atrial fibrillation/flutter, atrioventricular (nodal) re-entrant tachycardias (AVRT/AVNRT), and ventricular arrhythmias], ablation site (left atrium, right atrium, ventricular, and other/unknown), ablation method (cryoablation, radiofrequency ablation, other), IBD subtype (CD vs. UC), sex (male vs. female), age group (20–49, 50–69, and ≥70 years), and the presence of pre-existing heart failure, coronary artery disease, diabetes mellitus, dyslipidaemia, and hypertension. For analyses by IBD subtype, re-matching was performed between individuals with CD and those with UC. ICD and procedure codes used to define covariates are provided in Supplementary material online, *Table S4*.

To explore the potential impact of healthcare utilization patterns, we additionally adjusted for the number of hospitalizations 18–6 months prior to the index catheter ablation. The 6-month window immediately preceding the ablation was chosen to avoid including hospitalizations potentially related to arrhythmia that subsequently led to catheter ablation.

As an additional analysis, the unmatched win ratio hierarchical test was used to compare each individual component of the composite outcome prioritizing more clinically relevant outcomes over less critical outcomes, such as hospitalization for arrhythmia. With this approach, every individual with IBD was compared with every individual in the propensity score–matched general population comparators. For the win ratio analysis, death was added as the highest-priority component of the hierarchical composite outcome to address competing risk bias.^17^ The hierarchical composite was defined from most to least important: (i) time to death; (ii) time to first reablation; (iii) time to first cardioversion; and (iv) time to first hospitalization for arrhythmia. All pairs were first compared by time to death; individuals with a longer time alive were considered the winners. If there was no winner, pairs were then compared by time to first reablation; again, individuals with a longer period of follow-up free of reablation were considered the winners. In the event of a tie for the first 2 tiers of the hierarchy (i.e. within the shorter follow-up time between participants in the match, none had died or undergone reablation), the pair was compared by cardioversion. Individuals with a longer follow-up free of cardioversion were considered winners. Finally, individuals were compared by time to first hospitalization for arrhythmia. Time-to-event comparisons were performed within the minimum follow-up time of each patient pair. The overall number of wins was divided by the number of losses for patients with IBD compared with general population comparators to calculate the win ratio. The win ratio was tested with a two-sided α level of 0.05.

Effect estimates and corresponding 95% confidence intervals (CIs) were derived using linear, logistic, or Cox proportional hazards regression models, as appropriate. The proportional hazards assumption was assessed by inspection of Schoenfeld residuals over time. Two-sided *P* < 0.05 were considered statistically significant. All statistical analyses were performed using STATA software (version 18.0).

## Results

After propensity score–matching, 251 adults with IBD and 2429 matched general population comparators were included (see Supplementary material online, *Figure S1*). Among patients with IBD, 117 (47%) had CD and 134 (53%) had UC. Patients with IBD were predominantly male (58%) and most patients were older than 50 years (7% aged 20–49 years, 51% 50–69 years, and 42% ≥ 70 years). Atrial catheter ablation was the most common procedure among patients with IBD (left atrium: 40%; right atrium: 46%), whereas ventricular and other indications accounted for 15% of procedures. The most frequent indications for catheter ablation were atrial fibrillation/flutter (66%), followed by AVNRT/AVRT (21%), ventricular arrhythmias (13%), and unknown indications (<1%). Radiofrequency ablation was performed in approximately three-quarters of patients with IBD, with cryoablation or other modalities used in the remainder. Overall, patients with IBD had a high burden of comorbidities. Additional baseline characteristics after propensity score–matching are presented in *Table 1* (and before matching in supplementary material online, *Table S5*).

**Table 1:** Baseline characteristics of the propensity score–matched cohorts

	Total	IBD	Matched comparators	SMD
	*N* = 2680	*N* = 251	*N* = 2429	
Sociodemographics, *n* (%)				
Age categories				0.03
20–49 years	259 (9.7)	18 (7.2)	241 (9.9)	
50–69 years	1301 (48.5)	127 (50.6)	1174 (48.3)	
≥70 years	1120 (41.8)	106 (42.2)	1014 (41.7)	
Female sex	1154 (43.1)	105 (41.8)	1049 (43.2)	0.03
Swiss resident	2482 (92.6)	230 (91.6)	2252 (92.7)	0.04
Hospital category				0.007
University	1205 (45.0)	115 (45.8)	1090 (44.9)	
Tertiary	1093 (40.8)	97 (38.6)	996 (41.0)	
Secondary	382 (14.3)	39 (15.5)	343 (14.1)	
Study period				0.03
2012–15	747 (27.9)	67 (26.7)	680 (28.0)	
2016–19	1039 (38.8)	98 (39.0)	941 (38.7)	
2020–22	894 (33.4)	86 (34.3)	808 (33.3)	
Procedural characteristics				
Indication for ablation				0.05
Atrial fibrillation/flutter	1810 (67.5)	165 (65.7)	1645 (67.7)	
AVNRT/AVRT	549 (20.5)	52 (20.7)	497 (20.5)	
Ventricular arrhythmias	292 (10.9)	32 (12.7)	260 (10.7)	
Unknown	29 (1.1)	2 (0.8)	27 (1.1)	
Ablation site				<0.001
Left atrium	1072 (40.0)	107 (42.6)	965 (39.7)	
Right atrium	1231 (45.9)	104 (41.4)	1127 (46.4)	
Ventricular	279 (10.4)	29 (11.6)	250 (10.3)	
Unknown	98 (3.7)	11 (4.4)	87 (3.6)	
Ablation procedure				0.01
Cryoablation	209 (7.8)	17 (6.8)	192 (7.9)	
Radiofrequency ablation	2051 (76.5)	197 (78.5)	1854 (76.3)	
Other	420 (15.7)	37 (14.7)	383 (15.8)	
Comorbidities				
Diabetes mellitus (type 1 and 2)	399 (14.9)	38 (15.1)	361 (14.9)	0.008
Hypertension	1551 (57.9)	152 (60.6)	1399 (57.6)	0.06
Dyslipidaemia	801 (29.9)	80 (31.9)	721 (29.7)	0.05
Obesity	226 (8.4)	23 (9.2)	203 (8.4)	0.03
Coronary artery disease	879 (32.8)	88 (35.1)	791 (32.6)	0.05
Congestive heart failure	936 (34.9)	91 (36.3)	845 (34.8)	0.03
Atrial fibrillation/flutter	1940 (72.4)	182 (72.5)	1758 (72.4)	0.003
Cerebrovascular disease	194 (7.2)	21 (8.4)	173 (7.1)	0.05
Peripheral artery disease	100 (3.7)	11 (4.4)	89 (3.7)	0.04
Chronic kidney disease	476 (17.8)	47 (18.7)	429 (17.7)	0.03
COPD	139 (5.2)	15 (6.0)	124 (5.1)	0.04
CCI, category				0.01
<1	966 (36.0)	90 (35.9)	876 (36.1)	
1	434 (16.2)	39 (15.5)	395 (16.3)	
≥2	1280 (47.8)	122 (48.6)	1158 (47.7)	

AVNRT, atrioventricular nodal re-entrant tachycardia; AVRT, atrioventricular re-entrant tachycardia; CCI, Charlson Comorbidity Index; COPD, chronic obstructive pulmonary disease; IBD, inflammatory bowel disease; IQR, interquartile range; SD, standard deviation; SMD, standardized mean difference.

### Adverse arrhythmia-related outcomes after catheter ablation

During a median follow-up of 2.6 years (IQR 0.8–5.6; maximum 11.0 years), 81 adverse arrhythmia-related events occurred among patients with IBD [32.3%, IR: 100.8/1000 person-years (PY)] and 640 among matched comparators (26.3%, IR: 73.3/1000 PY) (*Figure 1A* and *Table 2*). Compared with propensity score–matched comparators, patients with IBD had a 30% higher hazard of the composite adverse arrhythmia-related outcome [HR: 1.30 (95% CI, 1.03–1.64)] (*Table 2*).

**Figure 1:**
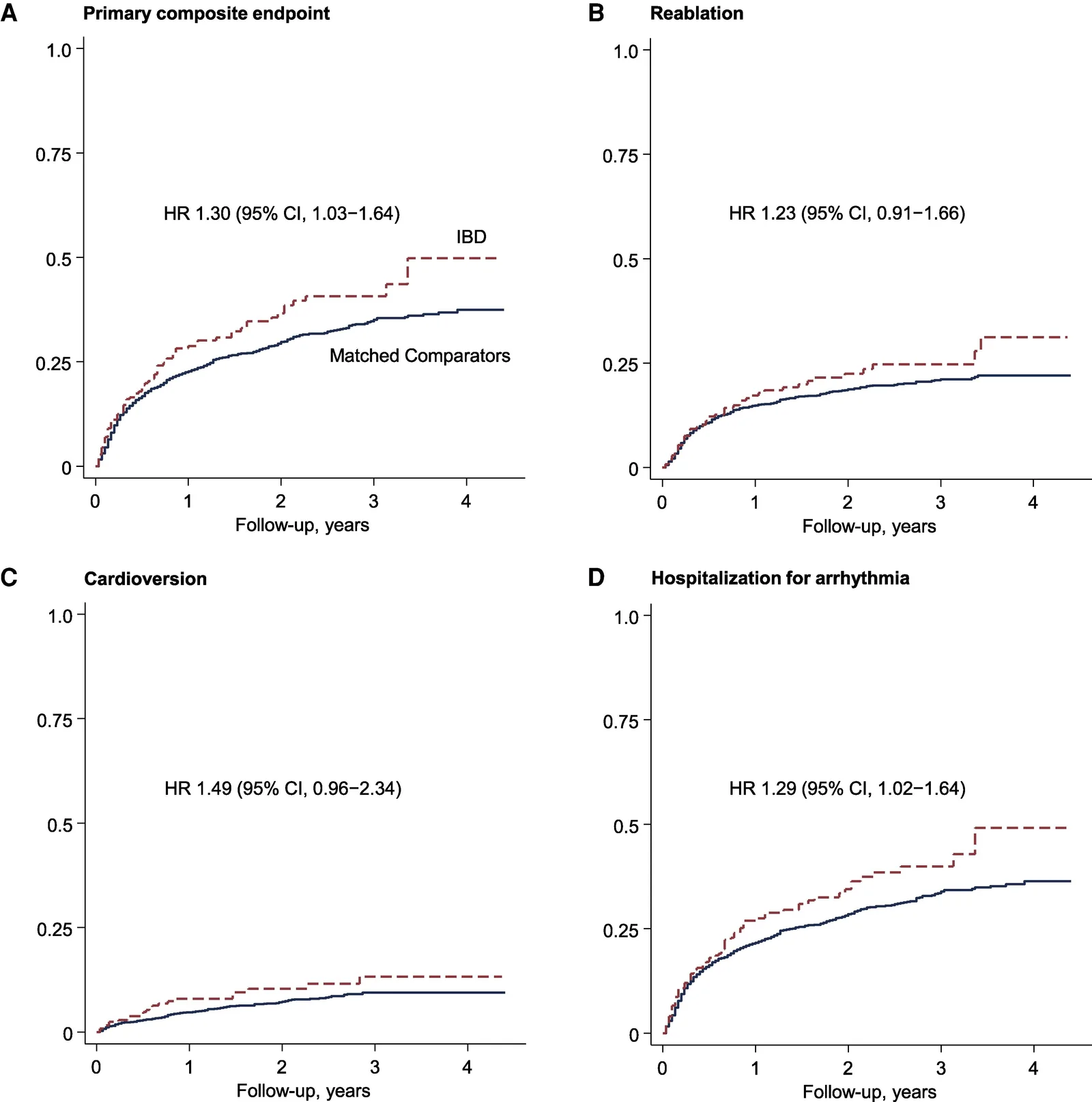
Kaplan–Meier curves and hazard ratios (HRs) of the composite primary endpoint of adverse arrhythmia-related outcomes (*A*) and individual components of the composite outcome (*B–D*), following catheter ablation for arrhythmia in patients with inflammatory bowel disease (IBD) and propensity score–matched general population comparators.

**Table 2:** Primary and secondary endpoints after catheter ablation in patients with inflammatory bowel disease compared to propensity score–matched comparators

	No. of events (IR, per 1000 PY)		Hazard ratio (95% CI)	*P*-value
	IBD	Matched comparators		
Primary endpoint				
Adverse arrhythmia-related outcomes Composite of reablation, cardioversion, or hospitalization for arrhythmia	81 (100.8)	640 (73.3)	1.30 (1.03–1.64)	0.03
Secondary endpoints				
Reablation	48 (52.9)	388 (41.3)	1.23 (0.91–1.66)	0.18
Cardioversion	22 (21.7)	151 (14.3)	1.49 (0.96–2.34)	0.08
Hospitalization for arrhythmia	78 (95.0)	615 (69.8)	1.29 (1.02–1.64)	0.04
4-point MACE^a^	62 (59.0)	532 (51.7)	1.14 (0.87–1.49)	0.35
Acute myocardial infarction	9 (8.5)	76 (7.0)	1.21 (0.61–2.43)	0.58
Stroke	5 (4.7)	39 (3.4)	1.37 (0.54–3.50)	0.51
Hospitalization for heart failure	26 (23.4)	244 (23.3)	1.00 (0.66–1.52)	0.99
All-cause mortality	44 (39.7)	339 (30.7)	1.29 (0.94–1.78)	0.11

^a^Four-point MACE was defined as a composite outcome measure of the first of any of acute myocardial infarction, stroke, hospitalization for heart failure, and all-cause mortality.

CI, confidence interval; HR, hazard ratio; MACE, major adverse cardiovascular events; PY, person-years.

Among individual components of the composite outcome, the hazard of hospitalization for arrhythmia was increased [HR 1.29 (95% CI, 1.02–1.64)], whereas reablation [HR 1.23 (95% CI, 0.91–1.66)] and cardioversion [HR 1.49 (95% CI, 0.96–2.34)] did not reach statistical significance (*Table 2* and *Figures 1B–D*).

### Non-arrhythmia-related cardiovascular and in-hospital outcomes after catheter ablation

The hazard of the 4-point MACE outcome was not increased among patients with IBD [1.14 (95% CI, 0.87–1.49)], and no individual MACE component differed significantly between groups (*Table 2*). In analyses of in-hospital outcomes, including ICU admission, length of ICU stay, intubation, duration of intubation, LOS, and all-cause readmission within 30 days, only the odds of 30-day readmission were higher among patients with IBD compared with matched comparators [odds ratio 2.05 (95% CI, 1.41–2.97)[ (*Table 3*).

**Table 3:** In-hospital outcomes after catheter ablation in patients with inflammatory bowel disease compared to propensity score–matched comparators

	*n* (%)/mean (SD)		OR (95% CI)	*P*-value^a^
Outcome	IBD	Matched comparators		
ICU admission rate	21 (8.4)	195 (8.0)	1.05 (0.65–1.67)	0.85
Length of ICU stay, d	0.4 (3.2)	0.3 (3.0)	0.06 (−0.34–0.45)	0.78
Intubation rate	10 (4.0)	73 (3.0)	1.34 (0.68–2.63)	0.40
Duration of intubation, h	7.5 (61.8)	4.9 (65.0)	2.66 (−7.69–13.00)	0.62
Length of hospital stay, d	3.7 (7.3)	3.5 (7.3)	0.19 (−0.76–1.15)	0.69
30-day readmission rate	39 (15.5)	200 (8.2)	2.05 (1.41–2.97)	<0.001

^a^Data on length of ICU stay, intubation, and length of hospital stay are presented as duration (SD) and regression coefficient (95%). All other outcomes are presented as odds ratios (OR).

d, days; h, hours; IBD, inflammatory bowel disease; ICU, intensive care unit; OR, odds ratio; SD, standard deviation.

### Stratified and sensitivity analyses

In stratified analyses by IBD subtype, the hazard of adverse arrhythmia-related outcomes was similar between the two subtypes [CD: HR 1.29 (95% CI, 0.92–1.81) vs. UC: HR 1.31 (95% CI, 0.96–1.78); *P* = 0.75] (*Figure 2*). When stratified by indication for the index ablation, atrial fibrillation/flutter was associated with a higher hazard of adverse arrhythmia-related outcomes [1.41 (95% CI, 1.08–1.83)]. No differences in hazards were observed across sex or age groups. Similarly, no effect modification was observed by pre-existing comorbidities, including heart failure, coronary artery disease, diabetes mellitus, dyslipidaemia, or hypertension (*Figure 2*). Risk estimates were further consistent across ablation modalities, including radiofrequency, cryoablation, and other techniques.

**Figure 2:**
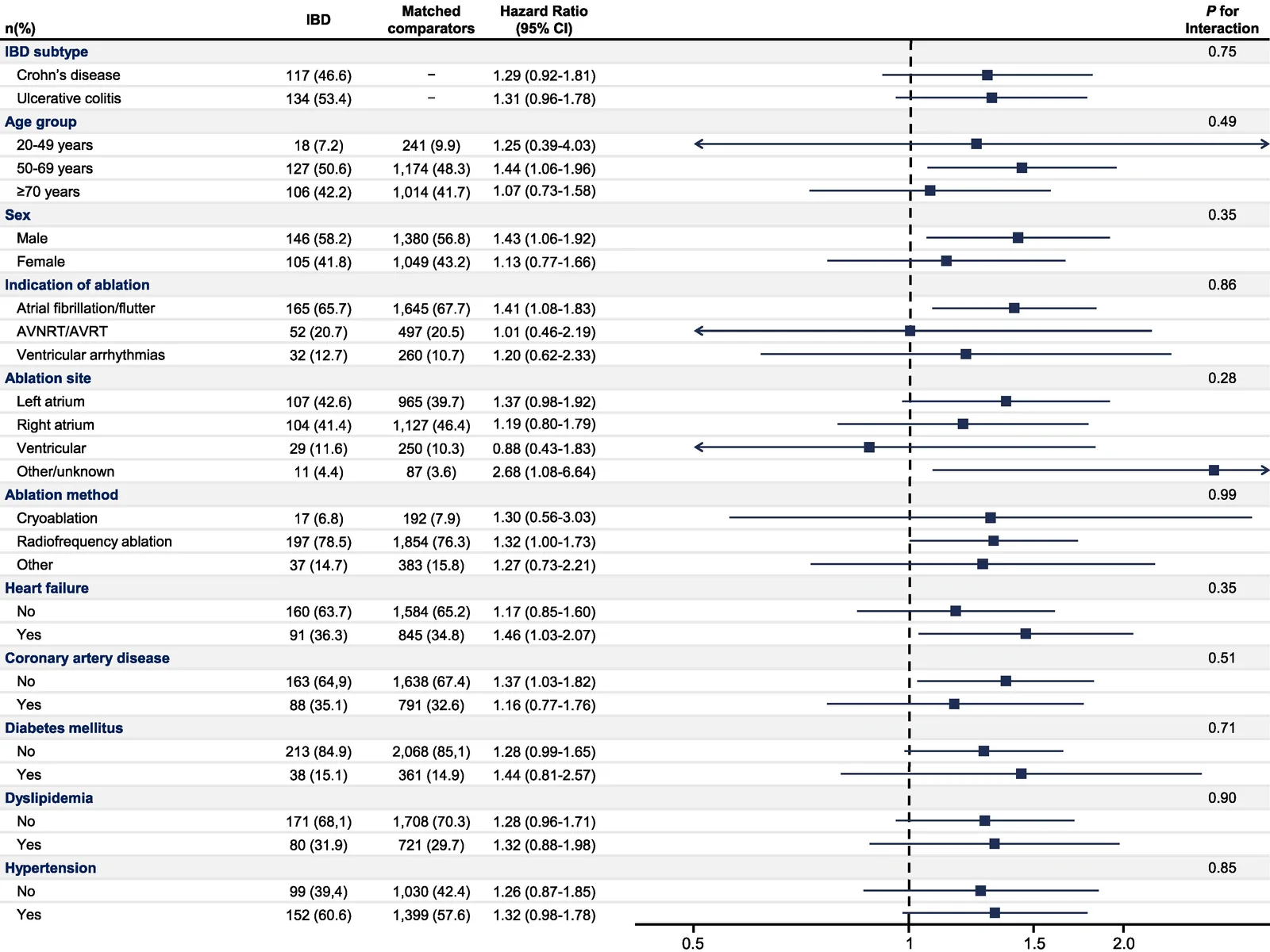
Stratified analyses of the composite outcome of adverse arrhythmia outcomes after catheter ablation in patients with inflammatory bowel disease (IBD) and propensity score–matched population comparators.

When additionally adjusting for the number of hospitalizations 18–6 months prior to the index catheter ablation, the overall results remained robust, with comparable HRs for the main composite outcome [HR 1.27 (95% CI, 1.00–1.60)] and the respective individual components [reablation: HR 1.22 (95% CI, 0.90–1.65), cardioversion: HR 1.47 (95% CI, 0.94–2.30), and hospitalization for arrhythmia: HR 1.26 (95% CI, 0.99–1.60)].

The win ratio for the hierarchical composite endpoint was 0.80 (95% CI, 0.65–0.99; *P* = 0.04), indicating that patients with IBD had 20% lower odds of favourable composite outcomes than matched comparators after ablation. The hierarchical win ratio analysis showed that the composite outcome was primarily driven by higher rates of cardioversion (component win ratio 0.41) and, to a lesser extent, by differences in death, reablation, and hospitalization for arrhythmia (*Table 4*). *Figure 3* illustrates the hierarchical flow of patient-pair comparisons at each outcome level.

**Figure 3:**
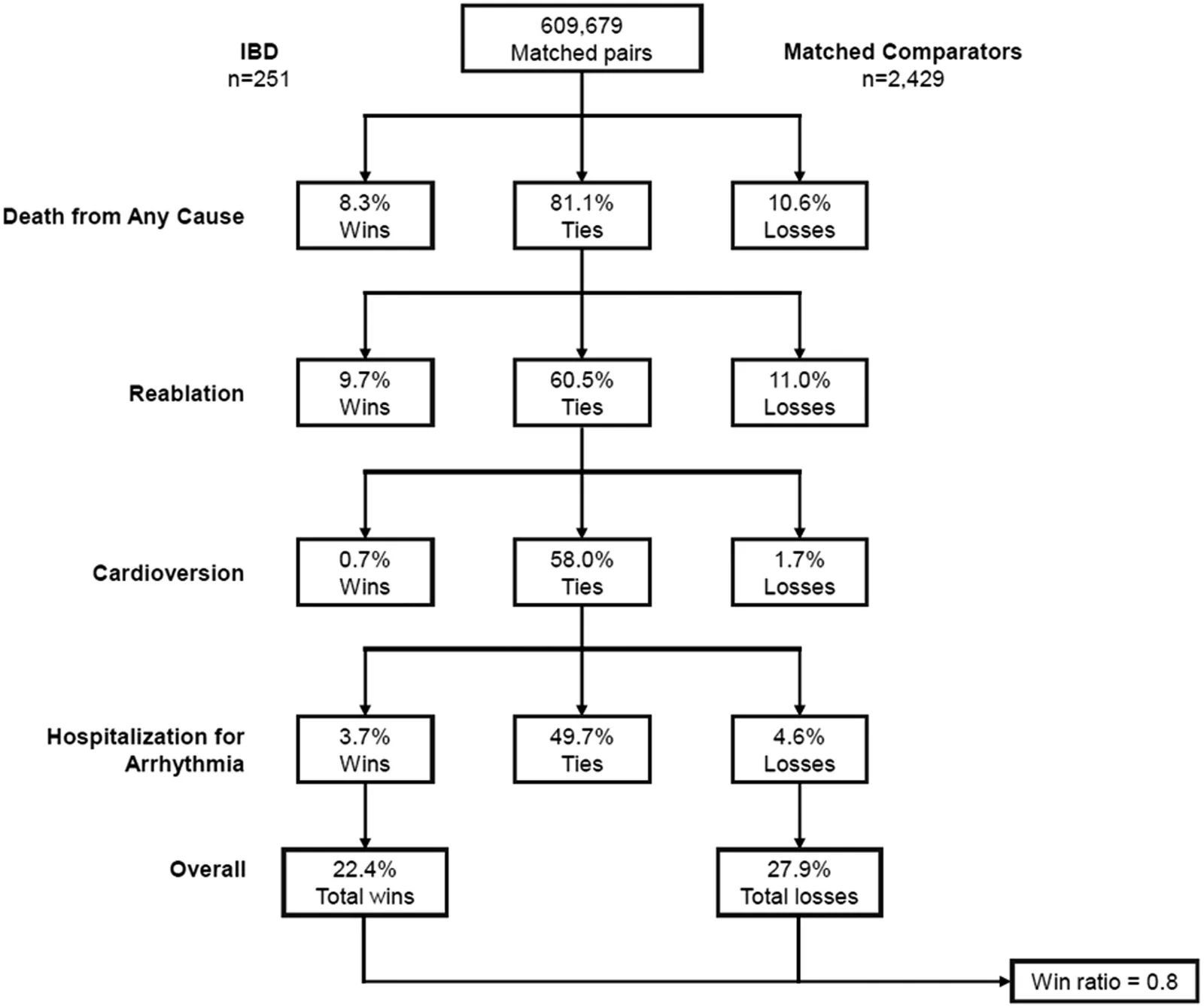
Flowchart of win ratio outcomes and components. Visual representation of the hierarchical composite outcome analysed using win ratio methodology for IBD vs. matched comparators. The hierarchical composite prioritized components as (i) death, (ii) reablation, (iii) cardioversion, and (iv) hospitalization for arrhythmia. The win ratio is calculated as total wins divided by total losses with values < 1 indicating worse composite outcomes in patients with IBD compared with matched comparators.

**Table 4:** Component contribution of the win ratio analysis

Hierarchical level	Total pairs evaluated	IBD wins	Ties	Comparator wins	Pairs proceeding to next level	Component win ratio
Starting pairs	609 679	–	–	–	609 679	–
1. Mortality	609 679	50 453 (8.3)	494 624 (81.1)	64 602 (10.6)	494 624	0.78
2. Reablation	494 624	58 984 (9.7)	368 610 (60.5)	67 030 (11.0)	368 610	0.88
3. Cardioversion	368 610	4277 (0.7)	353 848 (58.0)	10 485 (1.7)	353 848	0.41
4. Hospitalization for arrhythmia	353 848	22 597 (3.7)	303 165 (49.7)	28 086 (4.6)	–	0.80
Overall	609 679	136 311 (22.4)	303 165 (49.7)	170 203 (27.9)	–	0.80

Tabular representation of the hierarchical composite outcome analysed using win ratio methodology for patients with inflammatory bowel disease (IBD) vs. matched comparators. The hierarchical composite prioritized components as (i) death, (ii) reablation, (iii) cardioversion, and (iv) hospitalization for arrhythmia. The win ratio is calculated as total wins divided by total losses with values < 1 indicating worse composite outcomes in patients with IBD compared with matched comparators. The table shows number (*n*) and percentage (%) unless else indicated.

## Discussion

In this nationwide Swiss propensity score–matched cohort study of 251 adults with IBD who underwent catheter ablation between 2012 and 2022, IBD was associated with 30% higher hazard of adverse arrhythmia-related outcomes compared with matched comparators, primarily driven by arrhythmia-related re-hospitalizations.

Previous studies have reported an ∼1.5-fold higher long-term risk of arrhythmia in patients with IBD,^6,18^ along with elevated risks of heart failure,^7^ ischaemic stroke,^19^ myocardial infarction,^5^ and adverse cardiovascular events following coronary interventions.^8^ However, outcomes following catheter ablation for arrhythmia in this population have not been previously examined. In the present study, IBD was associated with 30% higher hazard of the composite adverse arrhythmia-related outcome compared with propensity score–matched population comparators. In secondary outcome analyses hazard ratios for all individual components were increased and ranged from 1.23 to 1.49, only hospitalization for arrhythmia reached statistical significance. The absence of statistical significance for reablation and electrical cardioversion is most likely due to limited power for these analyses.

To compare each individual component of the composite outcome, while prioritizing clinically more relevant over less critical outcomes such as hospitalization for arrhythmia, we performed hierarchical win ratio analysis. The win ratio analysis in our study provides important mechanistic insight into the composite endpoint findings, indicating that the increased risk in IBD patients is primarily driven by cardioversion rather than non-specific hospitalizations. While Cox regression demonstrated statistical significance only for hospitalization for arrhythmia among the individual components, the hierarchical win ratio approach clarifies the clinical nature of these events. Cardioversion showed the strongest component effect (component win ratio 0.41), indicating that IBD patients experienced substantially more arrhythmia recurrences requiring acute rhythm control interventions than matched comparators.

Patients undergoing catheter ablation for atrial fibrillation/flutter had a higher hazard of the composite of adverse arrhythmia-related outcomes (HR 1.41). Consistent with this finding, left atrial ablation was associated with a numerically higher hazard, although this difference did not reach statistical significance. These observations may reflect the potential influence of systemic inflammation in IBD on cardiac electrophysiology, including the development of myocardial fibrosis as a substrate for arrhythmias. Supporting this hypothesis, a recent cardiac magnetic resonance study using myocardial tissue phenotyping demonstrated a higher prevalence of myocardial fibrosis and adverse left ventricular remodelling in patients with active IBD compared with individuals without IBD—despite no prior diagnosis of heart failure or inflammatory heart disease.^20^ Fibrotic remodelling is a well-recognized feature of IBD, particularly in CD, where intestinal fibrosis and fibrostenotic disease are common.^21^ It is tempting to speculate whether extraintestinal fibrosis in the myocardium might contribute to the observed arrhythmogenic risk and higher recurrence risk.

In analyses of in-hospital outcomes, patients with IBD had ∼2-fold higher odds of 30-day readmission compared with matched population comparators. This finding is consistent with the increased risk of arrhythmia-related hospitalization observed during follow-up and aligns with prior evidence of higher 30-day readmission rates among patients with IBD after coronary interventions.^8^ Collectively, these observations suggest that patients with IBD undergoing catheter ablation may require closer post-procedural monitoring and follow-up to prevent unplanned hospitalizations and re-interventions.

As expected, in-hospital outcomes—including ICU admission, length of ICU stay, intubation rates, duration of intubation, and overall length of stay—did not differ between patients with and without IBD. These findings indicate that the short-term procedural safety of catheter ablation remains robust in the IBD population, comparable to that of non-IBD controls and unlikely to be substantially influenced by systemic inflammation.

To explore the potential impact of healthcare utilization patterns, we additionally adjusted for the number of hospitalizations 18–6 months prior to the index catheter ablation. Overall, the results remained robust, suggesting that our findings are unlikely to be explained solely by differential healthcare utilization patterns.

Although the observational and explorative nature of our study does not allow us to draw causal inferences or provide mechanistic explanations for our findings, we hypothesize that the observed association between IBD and adverse arrhythmia-related outcomes is likely multifactorial, involving mechanisms related to systemic inflammation and patient-specific factors. Systemic inflammation has long been implicated in the development of coronary artery disease and arrhythmias,^4,22^ and similar inflammatory pathways have been proposed in IBD.^23–25^ Although the mechanisms underlying arrhythmogenesis in IBD are not fully understood, oxidative stress, autonomic dysfunction, myocardial fibrosis, and electrolyte imbalances have been proposed as contributing factors, potentially mediated by chronic systemic inflammation.^9–13^ Moreover, although speculatively, alterations in the gut microbiota and increased intestinal permeability in IBD may facilitate the translocation of microbial metabolites and inflammatory mediators into the circulation, thereby influencing cardiac electrophysiology through immune, metabolic, and neural pathways.^26,27^

Inflammatory bowel disease typically follows a relapsing–remitting disease course, with periods of active inflammation interspersed with remission. Episodes of disease activity may be associated with an increased risk of arrhythmia and cardiovascular events. In support of this, a nationwide Danish registry study reported higher rates of recurrent arrhythmia during periods of IBD flares or active disease.^28^ These observations highlight the potential importance of effective disease control in IBD to mitigate the excess cardiovascular risk.

Although our findings are novel and require confirmation in future studies, we believe that they give rise to several important potential clinical considerations. First, the consistently reported excess cardiovascular risk in patients with IBD may necessitate a careful pre-procedural risk–benefit assessment. Clinicians should carefully weigh the indications for catheter ablation against individual comorbidities and the current inflammatory burden. Second, if confirmed in future studies, our results indicate a potential importance of pre-procedural risk stratification and structured post-ablation surveillance in patients with IBD. Third, given the systemic inflammation in IBD and the trend towards increased early hospitalizations for arrhythmia observed in our study, the role of adjunctive antiarrhythmic or anti-inflammatory drug therapy during the blanking period may merit consideration. Short-term antiarrhythmic therapy has been shown to reduce early recurrences after catheter ablation and may be particularly relevant in patients with heightened inflammatory activity.^29^ Furthermore, the role of cardioprotective agents with anti-inflammatory properties, such as colchicine or statins, remains a promising area for future dedicated investigation in IBD cohorts.^30–38^ Fifth, gastroenterologists must weigh the cardiovascular risks of advanced IBD therapies against their benefits in controlling intestinal inflammation and thereby reducing cardiovascular complications. Finally, these challenges highlight the necessity of a multidisciplinary ‘Heart-Gut’ collaboration. Integrated care pathways involving both cardiologists and gastroenterologists are essential to optimize intestinal disease control, refine peri-interventional management, and ensure timely cardiovascular follow-up.

The strengths of this study include its nationwide, population-based design, which is expected to ensure high validity of exposure and outcome definitions. Comprehensive national inclusion minimizes selection bias and enhances external validity. In addition, the propensity score–matched design provides robust, real-world estimates of patient-centred hospital outcomes among adults with IBD undergoing routine catheter ablation care.

Our study has some limitations. First, misclassification of IBD cannot be fully excluded, as patients with undiagnosed IBD may have been included among matched comparators, and the IBD diagnosis has not been formally validated in the Swiss inpatient registry. Nevertheless, given the extensive diagnostic work-up typically required for IBD, our case definition is robust, and prior validation studies in Swedish and US inpatient registries using similar ICD-10-based definitions have reported predictive values exceeding 90%.^39,40^ Second, data on lifestyle factors and clinical characteristics, including body mass index, alcohol consumption, smoking status, diet, and physical activity, were unavailable, and residual confounding related to these factors cannot be fully excluded. However, propensity score–matching incorporated multiple comorbidities linked to lifestyle, partially mitigating this limitation. Third, information on IBD disease activity, IBD treatment exposure, and biomarkers of active disease was unavailable, precluding assessment of IBD disease severity or treatment-specific effects. Fourth, information on arrhythmia burden, AF subtype, procedural characteristics, lesion sets, operator and centre experience, antiarrhythmic therapy, and anticoagulation strategies was not available, further limiting the granularity of the analyses and potentially confounding the observed associations. Despite these limitations, the nationwide design and real-world inpatient data provide important insights into arrhythmia-related outcomes after catheter ablation among unselected patients with IBD with virtually no loss to follow-up.

## Conclusion

In this nationwide propensity score–matched cohort study, patients with IBD experienced a statistically significant increase in adverse arrhythmia-related outcomes following catheter ablation compared with matched individuals without IBD. This excess risk was primarily driven by a higher rate of hospitalizations for arrhythmia during follow-up, while rates of reablation and cardioversion were numerically higher but did not reach statistical significance.

## Supplementary Material

oeag168_Supplementary_Data

## Data Availability

The data analysed in this study is subject to the following licences/restrictions: the data that support the findings of this study are available from the Swiss Federal Office for Statistics. Restrictions apply to the availability of these data, which were used under licence for this study. Requests to access these datasets should be directed to Swiss Federal Office for Statistics.
