# Supplementary material for: Adverse arrhythmia-related outcomes after catheter ablation in patients with inflammatory bowel disease: a nationwide propensity score–matched cohort study
Source: Eur Heart J Open. 2026 Oct 8;6(6):oeag168. doi: 10.1093/ehjopen/oeag168 (PMC13739052; doi:10.1093/ehjopen/oeag168)
Supplement: Supplementary file 1 [file oeag168_supplementary_data.docx]

Supplementary Appendix

The authors have provided this appendix to give readers additional information about their work:

**Adverse Arrhythmia-Related Outcomes After Catheter Ablation in Patients with Inflammatory Bowel Disease: A Nationwide Propensity Score–Matched Cohort Study**

Ramin Ebrahimi, Shaojie Chen, Davide Di Vece, Michele Maremmani, Ali Mahdi, Bahira Shahim, Jan Hendrik Niess, Alexander Kutz, Fahim Ebrahimi* and Anders Forss*

**These authors contributed equally.*

Table of Contents

[Supplementary Figures	3](#_Toc216457224)

[Figure S1. Flowchart of study population	3](#_Toc216457225)

[Figure S2. Propensity score distribution before and after matching	4](#_Toc216457226)

[Supplementary Tables	5](#_Toc216457227)

[Table S1. Procedure codes for catheter ablation procedures	5](#_Toc216457228)

[Table S2. International Classification of Disease (ICD) codes for inflammatory bowel disease	5](#_Toc216457229)

[Table S3. Definition of the primary and secondary outcomes	6](#_Toc216457230)

[Table S4. Comorbidities	7](#_Toc216457231)

[Table S5. Baseline characteristics before propensity score-matching	8](#_Toc216457232)

# Supplementary Figures

## Figure S1. Flowchart of study population

**44,204**

Individuals undergoing catheter ablation for arrhythmia

**1:10 Propensity score-matching**

**2,429**

Matched comparators without IBD

**251**

Patients with IBD

(117 with CD)

(134 with UC)

**41,524**	Excluded without diagnosis of IBD	and not eligible for matching

Flowchart of included patients and propensity score-matched comparators from the general population.

CD, Crohn’s disease; IBD, inflammatory bowel disease; UC, ulcerative colitis.

## Figure S2. Propensity score distribution before and after matching

Propensity score distribution of individuals with IBD and comparators (A) before and (B) after matching.

IBD, inflammatory bowel disease.

# Supplementary Tables

## Table S1. Procedure codes for catheter ablation procedures

|  | **Procedure code** |
| --- | --- |
| **Interventional ablation of cardiac arrhythmia** | 37.34.XX |

## Table S2. International Classification of Disease (ICD) codes for inflammatory bowel disease

|  | **ICD-10** |
| --- | --- |
| **Inflammatory bowel disease** | K50; K51 |
| **Crohn’s disease** | K50 |
| **Ulcerative colitis** | K51 |

## Table S3. Definition of the primary and secondary outcomes

| **Primary endpoint** | **Variable / ICD-10 / Procedure code** |
| --- | --- |
| **Composite of adverse arrhythmia outcomes** |  |
| Reablation | Procedure code: 37.34.XX |
| Cardioversion | Procedure codes: 99.61.XX; 99.62.XX |
| Hospitalization for arrhythmia | I48; I44.1; I44.2; I45.2; I45.3; I45.5; I45.6; I45.9; I46.0; I46.1; I46.9; I47.0; I47.1; I47.2; I49.0; R96.0 |
| **Secondary endpoints** |  |
| **4-point MACE** |  |
| Acute myocardial infarction | I21; I200 |
| Stroke | I60; I61; I63; I64 |
| Hospitalization for heart failure | I110; I50 |
| All-cause mortality | Death variable |
| **Length of hospital stay** | Days of hospitalization |
| **Intensive care admission rate** | Hours on intensive care unit |
| **Intubation rate** | Hours of intubation |

## Table S4. Comorbidities

|  | **ICD-10** |
| --- | --- |
| **Diabetes mellitus** | E10-E14 |
| **Hypertension** | I10-I16 |
| **Dyslipidemia** | E75, E77, E78 |
| **Obesity** | E65-E66 |
| **Coronary artery disease (CAD)** | I20-I25 |
| **Left main stem disease** | I25.14 |
| **Congestive heart failure** | I11.0; I13.0; I13.2; I25.5; I42.0; I42.6; I42.7; I42.8; I42.9; I43; I50 |
| **Atrial fibrillation/flutter** | I48 |
| **Cerebrovascular disease** | G45; I60; I62; I63; I64; I67; I69 |
| **Peripheral artery disease** | I70; I71; I731; I73.8 ; I73.9; I77.1; I79.0 ; I79.2; K55 |
| **Chronic kidney disease (CKD)** | N18-23; N26; T82.4; Y84.1; Q61; Z49; Z99.2; Z94.0 |
| **Chronic obstructive pulmonary disease (COPD)** | J40-47 |

## Table S5. Baseline characteristics before propensity score-matching

|  | **Total** | **IBD** | **Comparators** | **SMD (%)** |
| --- | --- | --- | --- | --- |
|  | **N=44,205** | **N=251** | **N=43,954** |  |
| **Sociodemographics, n(%)** |  |  |  |  |
| **Age categories** |  |  |  | 0.17 |
| 20-49 years | 5,939 (13.4) | 18 (7.2) | 5,921 (13.5) |  |
| 50-69 years | 22,007 (49.8) | 127 (50.6) | 21,880 (49.8) |  |
| ≥70 years | 16,259 (36.8) | 106 (42.2) | 16,153 (36.7) |  |
| **Female sex** | 16,065 (36.3) | 105 (41.8) | 15,960 (36.3) | 0.11 |
| **Swiss resident** | 38,993 (88.2) | 230 (91.6) | 38,763 (88.2) | 0.11 |
| **Hospital category** |  |  |  | 0.15 |
| University | 17,207 (38.9) | 115 (45.8) | 17,092 (38.9) |  |
| Tertiary | 18,413 (41.7) | 97 (38.6) | 18,316 (41.7) |  |
| Secondary | 8,585 (19.4) | 39 (15.5) | 8,546 (19.4) |  |
| **Study period** |  |  |  | 0.002 |
| 2012-2015 | 12,387 (28.0) | 67 (26.7) | 12,320 (28.0) |  |
| 2016-2019 | 16,169 (36.6) | 98 (39.0) | 16,071 (36.6) |  |
| 2020-2022 | 15,649 (35.4) | 86 (34.3) | 15,563 (35.4) |  |
| **Procedural characteristics** |  |  |  |  |
| **Indication for ablation** |  |  |  | 0.14 |
| Atrial fibrillation/flutter | 31,387 (71.0) | 165 (65.7) | 31,222 (71.0) |  |
| AVNRT/AVRT | 8,799 (19.9) | 52 (20.7) | 8,747 (19.9) |  |
| Ventricular arrhythmias | 3,617 (8.2) | 32 (12.7) | 3,585 (8.2) |  |
| Unknown | 402 (0.9) | 2 (0.8) | 400 (0.9) |  |
| **Ablation site** |  |  |  | 0.08 |
| Left atrium | 19,724 (44.6) | 107 (42.6) | 19,617 (44.6) |  |
| Right atrium | 19,205 (43.4) | 104 (41.4) | 19,101 (43.5) |  |
| Ventricular | 3,590 (8.1) | 29 (11.6) | 3,561 (8.1) |  |
| Unknown | 1,686 (3.8) | 11 (4.4) | 1,675 (3.8) |  |
| **Ablation method** |  |  |  | 0.06 |
| Cryoablation | 3,447 (7.8) | 17 (6.8) | 3,430 (7.8) |  |
| Radiofrequency ablation | 33,016 (74.7) | 197 (78.5) | 32,819 (74.7) |  |
| Other | 7,742 (17.5) | 37 (14.7) | 7,705 (17.5) |  |
| **Comorbidities** |  |  |  |  |
| Diabetes mellitus (type 1 and 2) | 4,669 (10.6) | 38 (15.1) | 4,631 (10.5) | 0.14 |
| Hypertension | 21,621 (48.9) | 152 (60.6) | 21,469 (48.8) | 0.24 |
| Dyslipidemia | 11,917 (27.0) | 80 (31.9) | 11,837 (26.9) | 0.11 |
| Obesity | 2,540 (5.7) | 23 (9.2) | 2,517 (5.7) | 0.13 |
| Coronary artery disease | 11,259 (25.5) | 88 (35.1) | 11,171 (25.4) | 0.21 |
| Congestive heart failure | 10,922 (24.7) | 91 (36.3) | 10,831 (24.6) | 0.25 |
| Atrial fibrillation/flutter | 33,367 (75.5) | 182 (72.5) | 33,185 (75.5) | 0.07 |
| Cerebrovascular disease | 2,317 (5.2) | 21 (8.4) | 2,296 (5.2) | 0.12 |
| Peripheral artery disease | 621 (1.4) | 11 (4.4) | 610 (1.4) | 0.18 |
| Chronic kidney disease | 4,214 (9.5) | 47 (18.7) | 4,167 (9.5) | 0.27 |
| COPD | 1,280 (2.9) | 15 (6.0) | 1,265 (2.9) | 0.15 |
| CCI, category |  |  |  | 0.46 |
| <1 | 24,031 (54.4) | 90 (35.9) | 23,941 (54.5) |  |
| ≥1 − <2 | 8,239 (18.6) | 39 (15.5) | 8,200 (18.7) |  |
| ≥2 | 11,935 (27.0) | 122 (48.6) | 11,813 (26.9) |  |

CCI, Charlson Comorbidity Index; COPD, chronic obstructive pulmonary disease; IBD, inflammatory bowel disease; IQR, interquartile range; SD, standard deviation; SMD, standardized mean difference
